# Supplementary material for: Tead1a Initiates Transcriptional Priming Through the TEAD1a/YAP‐Notch1‐Spi1/Cebpα Axis to Promote Neutrophil Fate
Source: Adv Sci (Weinh). 2025 Aug 28;12(41):e05441. doi: 10.1002/advs.202505441 (PMC12591127; doi:10.1002/advs.202505441)
Supplement: Supplementary file 4 — Supplementary Dataset [file ADVS-12-e05441-s004.zip › 3-2.zip!/3-2/02-2.cluster/cluster5/Enrich/ego_BP.pdf]

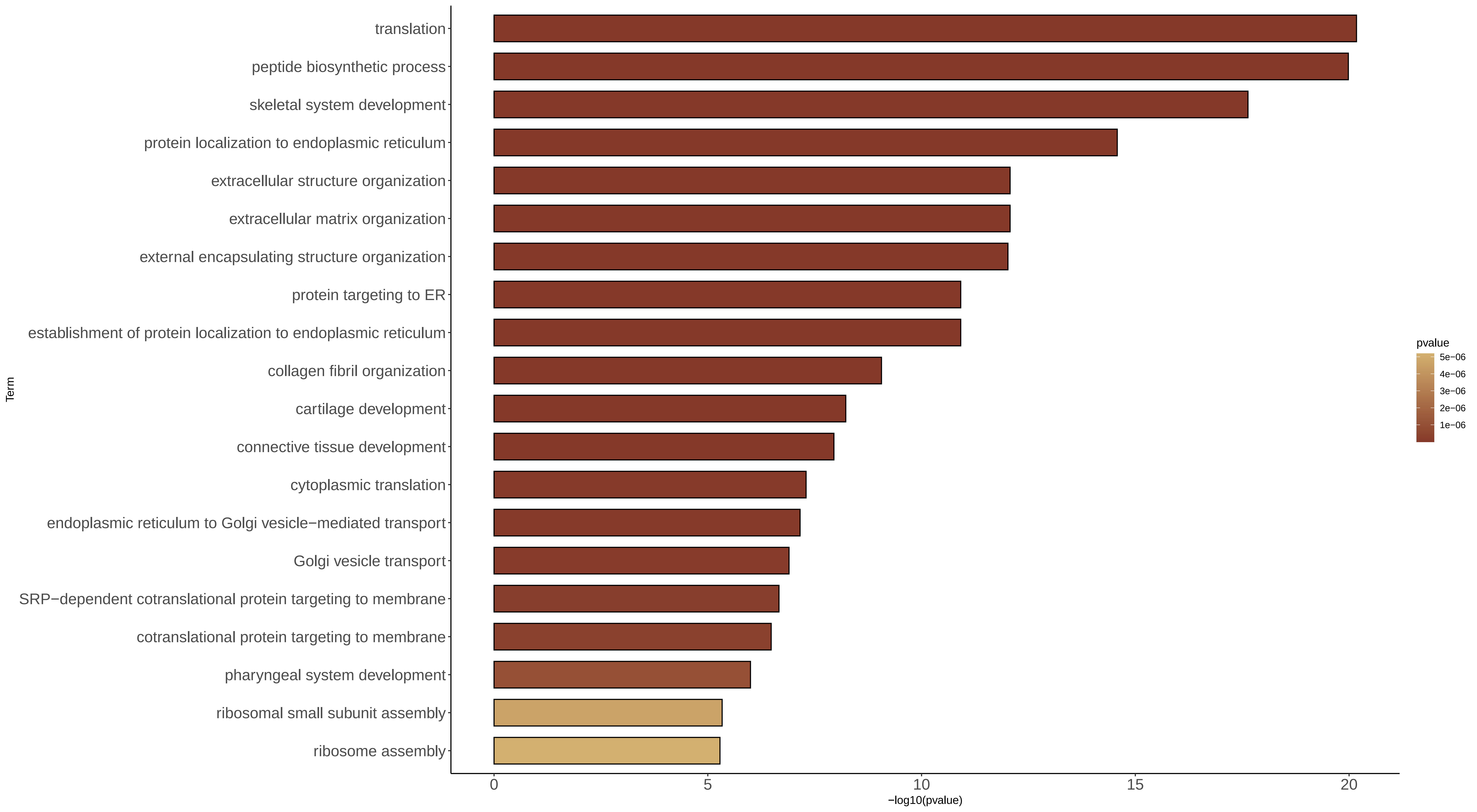
translation
peptide biosynthetic process
skeletal system development
protein localization to endoplasmic reticulum
extracellular structure organization
extracellular matrix organization
external encapsulating structure organization
protein targeting to ER
establishment of protein localization to endoplasmic reticulum
collagen fibril organization
cartilage development
connective tissue development
cytoplasmic translation
endoplasmic reticulum to Golgi vesicle−mediated transport
Golgi vesicle transport
SRP−dependent cotranslational protein targeting to membrane
cotranslational protein targeting to membrane
pharyngeal system development
ribosomal small subunit assembly
ribosome assembly
Term
0
5
10
15
20
−log10(pvalue)
pvalue
5e−06
4e−06
3e−06
2e−06
1e−06
